# *Mycobacterium tuberculosis *interactome analysis unravels potential pathways to drug resistance

**DOI:** 10.1186/1471-2180-8-234

**Published:** 2008-12-23

**Authors:** Karthik Raman, Nagasuma Chandra

**Affiliations:** 1 Bioinformatics Centre, Supercomputer Education and Research Centre Indian Institute of Science, Bangalore, 560 012, India

## Abstract

**Background:**

Emergence of drug resistant varieties of tuberculosis is posing a major threat to global tuberculosis eradication programmes. Although several approaches have been explored to counter resistance, there has been limited success due to a lack of understanding of how resistance emerges in bacteria upon drug treatment. A systems level analysis of the proteins involved is essential to gain insights into the routes required for emergence of drug resistance.

**Results:**

We derive a genome-scale protein-protein interaction network for *Mycobacterium tuberculosis *H37Rv from the STRING database, with proteins as nodes and interactions as edges. A set of proteins involved in both intrinsic and extrinsic drug resistance mechanisms are identified from literature. We then compute shortest paths from different drug targets to the set of resistance proteins in the protein-protein interactome, to derive a sub-network relevant to study emergence of drug resistance. The shortest paths are then scored and ranked based on a new scheme that considers (a) drug-induced gene upregulation data, from microarray experiments reported in literature, for the individual nodes and (b) edge-hubness, a network parameter which signifies centrality of a given edge in the network. High-scoring paths identified from this analysis indicate most plausible pathways for the emergence of drug resistance. Different targets appear to have different propensities for four drug resistance mechanisms. A new concept of 'co-targets' has been proposed to counter drug resistance, co-targets being defined as protein(s) that need to be simultaneously inhibited along with the intended target(s), to check emergence of resistance to a given drug.

**Conclusion:**

The study leads to the identification of possible pathways for drug resistance, providing novel insights into the problem of resistance. Knowledge of important proteins in such pathways enables identification of appropriate 'co-targets', best examples being RecA, Rv0823c, Rv0892 and DnaE1, for drugs targeting the mycolic acid pathway. Insights obtained about the propensity of a drug to trigger resistance will be useful both for more careful identification of drug targets as well as to identify target-co-target pairs, both implementable in early stages of drug discovery itself. This approach is also inherently generic, likely to significantly impact drug discovery.

## Background

Tuberculosis (TB) has remained one of the largest killer infectious diseases despite the availability of several chemotherapeutic agents and a vaccine [1]. The global burden of TB has taken a new dimension in the recent years due to the emergence of drug resistant varieties of *Mycobacterium tuberculosis*, besides synergy with HIV [2]. Global surveillance indicates that multi-drug resistant (MDR-TB) and extensively drug resistant TB (XDR-TB) are spreading to many countries, posing a major threat to TB eradication programmes [3,4]. Several different strategies are being explored to counter the problem of resistance, which include rotation of antibiotic combinations, enhanced medical supervision to ensure patient compliance, identification of new targets that may be less mutable, search for new chemical entities for known targets, use of virulence factors as targets and 'phenotypic conversion', which aims to inhibit the resistance mechanism employed by the bacterium [5]. While each of these may be very important measures, available statistics indicate that resistant forms are still on the rise, warranting more research in the area. Of the different measures listed, the most cogent in its approach in the long term, is targeting the resistance mechanisms, since it enables confronting the problem at its source. However, in order to use this strategy effectively, it is at the outset, essential to understand the ways by which resistance can emerge upon exposure to a given drug.

Studies on the molecular mechanisms of resistance to first-line and second-line anti-tubercular drugs have led to mapping of several mutations in the drug targets and the regulatory gene segments [3]. Besides these, the activation of the efflux pumps and drug-modifying enzymes are other known mechanisms of drug resistance [6]. Several studies in other organisms have reported the acquisition of drug-inactivating genes through horizontal gene transfer (HGT) as a means of selection of the resistant variety [7]. It is clear from these that diverse mechanisms can exist for generating resistance and that the proteins involved in each can be quite remote from the drug targets in terms of their functional classes. While some of these may result from the binding of the drugs directly to the relevant proteins such as cytochromes, or the transporter proteins, a majority of them cannot obviously be rationalised in the same manner. This is particularly true for resistance mechanisms such as mutations in the target or for acquisition of new genes through HGT to incapacitate the drug. This strongly indicates that communication mechanisms must exist in the cell, through which the required information reaches the appropriate components of the resistance machinery. The observed drug-induced expression data from microarray experiments too suggest that variations in expression pattern are quite complex and exhibit modifications in expression levels of several seemingly unrelated genes.

Knowledge of the molecular basis by which information flows from the specific drug target to the proteins elsewhere in the system, relevant to drug resistance, will help us address the issue of resistance in more systematic, rational and novel ways. With the availability of many genome-scale data from several studies, it is now feasible to address the issue of resistance from a systems perspective. Here, we use a proteome-scale network of protein-protein associations to discover possible pathways that may be responsible for generating drug resistance. The network analyses reported here further help in classification of these paths based on known resistance mechanisms. The study also identifies controlling hubs within these paths and suggests proteins that could be explored for their use as drug 'co-targets'.

## Results and discussion

**Figure 1:**
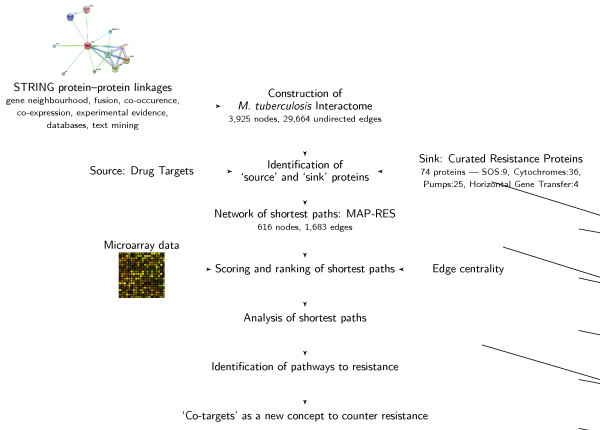
**Progression of experiments in this study**. A flowchart illustrating the progression of experiments in this study. Different aspects indicated in this are interactome construction, curation of the resistance proteins, identification of source and sink nodes, derivation of the MAP-RES network, incorporation of drug-induced gene upregulation data, scoring and ranking of paths, identification of pathways to drug resistance and finally the identification of co-targets as a new concept to counter drug resistance.

The different steps involved in this study are illustrated in the flowchart in Fig. 1. Interactions among proteins of *M. tuberculosis*, discerned from the STRING database, have been used to construct a protein-protein interactome, which enables a novel formulation of the problem of drug resistance and forms a first step towards countering drug resistance at the drug discovery stage itself. In particular, the questions addressed here are: (i) can we obtain insights into the possible routes through which information in the form of structural and biochemical signals can flow from the drug target(s) of a given drug to the molecular components of the resistance machinery, (ii) do different drugs follow different pathways of resistance, therefore triggering different resistance mechanisms, (iii) do different drugs have different propensities for inducing resistance and (iv) lastly, can we design intelligent 'road-blocks' to prevent the emergence of drug resistance.

### Interactome network

A proteome-scale interaction network of proteins in *M. tuberculosis *H37Rv was derived from the STRING database [8], which includes interactions from published literature describing experimentally studied interactions, as well as those from genome analysis using several well-established methods based on domain fusion, phylogenetic profiling and gene neighbourhood concepts. Thus, the network captures different types of interactions such as (a) physical complex formation between two proteins required to form a functional unit, (b) genes belonging to a single operon or to a common neighbourhood, (c) proteins in a given metabolic pathway and hence influenced by each other, (d) proteins whose associations are suggested based on predominant co-existence, co-expression, or domain fusion. This network represents a first comprehensive view of the connectivity among the various proteins, analogous to obtaining the road map of a city. In STRING, a confidence score is assigned to each identified protein-protein association, derived by benchmarking the performance of the predictions against a common reference set of trusted, true associations, which also takes into account the frequency or reciprocality of the detection [8,9]. After assignment of association scores and transfer between species, a final 'combined score' between any pair of proteins is assigned. It is computed under the assumption of independence for the various sources, in a naïve Bayesian fashion [8,9]. A higher score is assigned when an association is supported by several types of evidence, thus expressing increased confidence. With the methodology currently available, it is inevitable for a network of this type to contain some false positives as well as false negatives. To minimise this problem, all interactions tagged as 'low-confidence' in the STRING database have been eliminated from this study. 131,043 interactions were observed for 3,958 proteins of *M. tuberculosis*; the coverage of the *M. tuberculosis *proteome is seen to be as high as 99%. Of the total 131,043 interactions, 11,425 were labelled as 'high-confidence', and 18,239 as 'medium-confidence'. Interactions in these two categories (covering 3,925 of the proteins) were considered in our analysis (listed in Additional File 1, along with the individual scores). The considered network, despite its shortcomings, provides an excellent framework for navigating through the proteome. It also allows for refinement of the network upon the availability of new experimental data.

### Derivation of MAP-RES: a sub-network to study drug resistance

The interactome network thus obtained contains 3,925 nodes (proteins), with 29,664 undirected edges (interactions) between them. The clustering coefficient of the network is 0.447. This high clustering coefficient is indicative of the high density of connections in the network.

#### Defining source and sink nodes

In order to study the portion of the large interactome relevant for drug resistance, it is first required to define the 'source' and the 'sink' nodes for the flow of information. A 'source' node in this case refers to the drug target, where a drug is known to definitely make an impact, while the sink nodes refer to the possible molecular components of the resistance machinery. Three of the drugs – isoniazid, ethionamide and isoxyl – are known to be inhibitors of mycolic acid biosynthesis, for which the 26 proteins of the mycolic acid pathway (MAP) [10] were used as source. It can be envisaged that upon inhibition of a protein in a given pathway, metabolic adjustments often occur so as to minimise the effect of inhibition on the particular protein [6]. In order to incorporate the effect of such adjustments, we have considered the whole pathway as the source rather than an individual protein. There are also reports in the literature that multiple proteins in the MAP may be targeted by some of these drugs [11], making it important to consider the pathway as a whole.

Known mechanisms relevant to resistance were classified into four types (a) efflux pumps, which transport drugs out of the cell, (b) cytochromes and other target-modifying enzymes that could cause potential chemical modification of drug molecules, (c) SOS-response and DNA replication leading to mutations in the gene or its regulatory region, (d) proteins involved in HGT to import a target modifying or detoxifying protein from its environment. Although HGT is not known to be an important mechanism in conferring drug resistance in *M. tuberculosis*, four genes present in the *M. tuberculosis *genome bear close similarity to well-established HGT genes in other organisms. The report by Smith and Romesberg [7] implicates these proteins in drug resistance and therefore, these have been included in our curated set. We curated a list of 74 genes in the *M. tuberculosis *genome based on these mechanisms from published literature (Table 1). This curated set of 'resistance proteins' were grouped together as 'sink'.

**Table 1:** Curated list of resistance proteins

**Antibiotic efflux pumps **[6,29]
PstB (Rv0933), Rv2686c, Rv2687c, Rv1688c, IniA (Rv0342), Mmr (Rv3065)
Rv3239c, Rv3728, Rv2846c, Rv1877, Rv2333c, Rv2459, Rv1410c, Rv1250, Rv1258c, Rv0783c, Rv1634, Rv0849
**Hypothetical efflux pumps **[29]
Rv0191, Rv0037c, Rv2456c, Rv2994
Antibiotic degrading enzymes* [6]
BlaC (Rv2068c)
Target-modifying enzymes* [30,31]
Erm37 (Rv1988), WhiB7 (Rv3197A)
**SOS and related genes **[7]
DnaE2 (Rv3370c), RuvA (Rv2593c), RecA (Rv2737c), RecB (Rv0630c), RecC (Rv0631c), RecD (Rv0629c), DnaE1 (Rv1547), PolA (Rv1629), LexA (Rv2720)

**Genes implicated in horizontal gene transfer **[7,32]
SecA1 (Rv3240c), SecA2 (Rv1821), Rv3659c, Rv3660c

**Cytochromes **[32]
CcdA (Rv0527), CcsA (Rv0529), CtaB (Rv1451), CtaC (Rv2200c), CtaD (Rv3043c), CtaE (Rv2193), CydA (Rv1623c), CydB (Rv1622c), CydC (Rv1620c), CydD (Rv1621c), Cyp121 (Rv2276), Cyp123 (Rv0766c), Cyp124 (Rv2266), Cyp125 (Rv3545c), Cyp126 (Rv0778), Cyp128 (Rv2268c), Cyp130 (Rv1256c), Cyp132 (Rv1394c), Cyp135A1 (Rv0327c), Cyp135B1 (Rv0568), Cyp136 (Rv3059), Cyp137 (Rv3685c), Cyp138 (Rv0136), Cyp139 (Rv1666c), Cyp140 (Rv1880c), Cyp141 (Rv3121), Cyp142 (Rv3518c), Cyp143 (Rv1785c), Cyp144 (Rv1777), Cyp51 (Rv0764c), DipZ (Rv2874), LldD1 (Rv0694), LldD2 (Rv1872c), QcrB (Rv2196), QcrC (Rv2194), SdhC (Rv3316)

*These proteins are antibiotic degrading and target modifying proteins, but since they are very few, have been included with the pumps for convenience.

##### MAP-RES network

**Figure 2:**
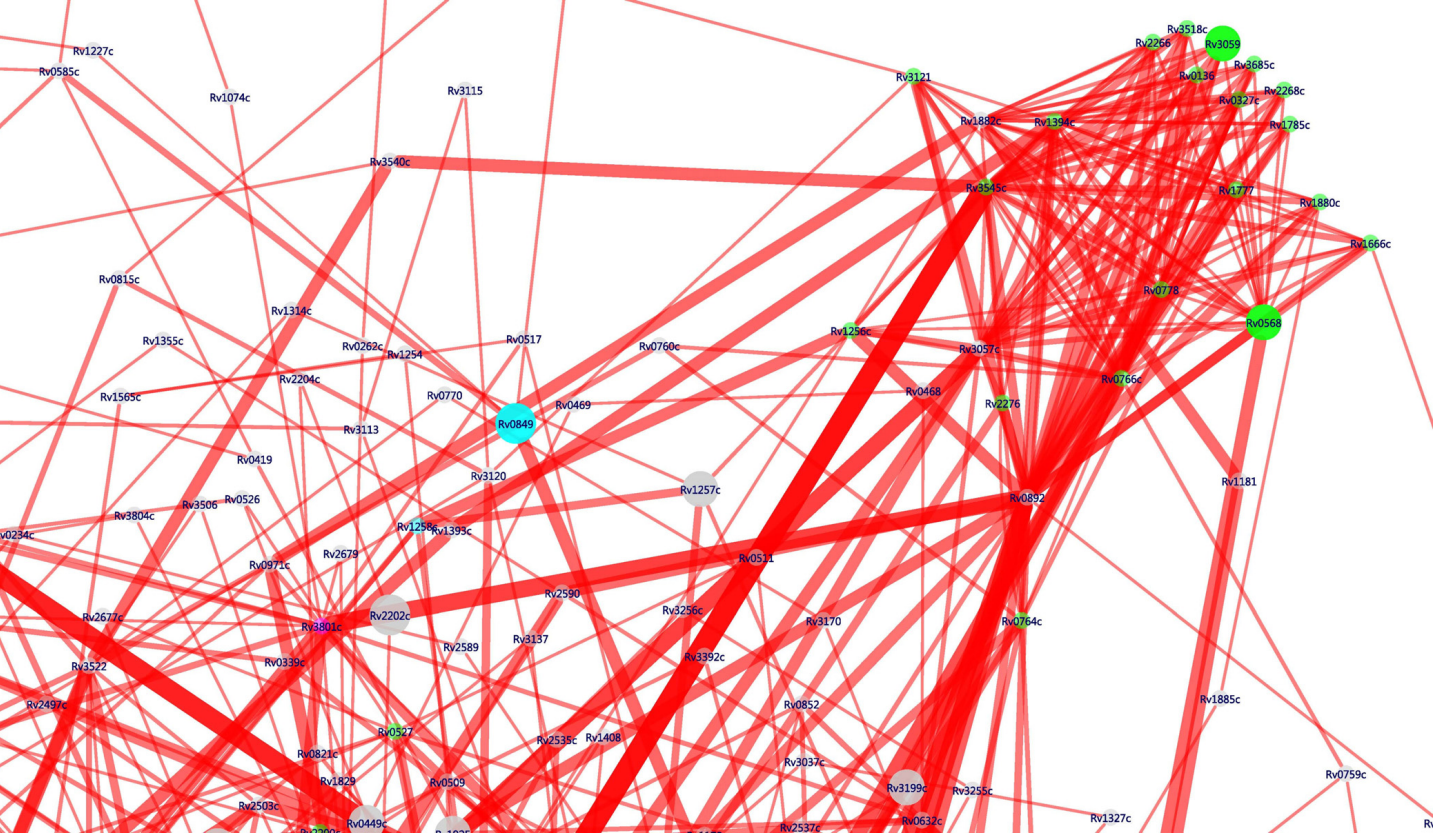
**Illustration of the MAP-RES network**. Illustration of a portion of the MAP-RES network. The full MAP-RES network (high-resolution zoomable PDF) is available as supplementary material [see Additional File 2]. Only a portion of it is shown here for clarity. In this, the tight clustering of the cytochrome proteins (coloured as green nodes) can be seen. Rv0892, a co-target suggested from this analysis links up the cytochrome clusters to the MAP proteins. Nodes correspond to the individual proteins in the network while the edges indicate interactions between them. Each class of nodes is coloured differently. The nodes are sized in proportion to the number of MAP drugs that induce its upregulation. The thickness of an edge is proportional to the number of times a shortest path is traversed through that edge.

Shortest 'paths' from source to the 'sink' (curated set of resistance proteins) were computed as described in the Methods section. A subset of the whole interactome network consisting only of the nodes and edges that form a part of the computed shortest paths was considered. This subset, consisting of 616 nodes and 1683 edges, which is referred to as the MAP-RES network hereafter (Fig. 2; also see Additional File 2), is a sub-network of the entire interactome described earlier. The clustering coefficient of the MAP-RES network is 0.056. The diameter of the network, indicating the maximum length of a shortest path in the network is 9. Of the 616 nodes in the network, 80 are upregulated in response to one or more drugs. For this sub-network, the number of neighbours for a node varies from one to 43. For example, for Rv0904c (AccD3), there are 41 neighbours; of these 41 interactions from STRING, 25 are high-confidence, and six of them have been corroborated through experimental studies. On the other hand, for Rv2459, a possible membrane transport protein, there is only one neighbour in the MAP-RES network.

#### Incorporating expression information

Microarray experiments have been reported in the literature, where variation in the expression of mycobacterial genes have been studied upon exposure to anti-tubercular drugs [12,13]. These experiments provide global views of the effects of anti-tubercular drugs on the mycobacterial proteome, identifying lists of genes whose expression levels were either increased or decreased upon exposure to the drugs. Such data were obtained for seven drugs, viz. isoniazid [12,13], ethionamide [13], isoxyl, tetrahydrolipstatin, SRI-221, SRI-967 and SRI-9190 [12]. The patterns of variation in terms of an increase or decrease in the expression levels of individual genes are complex, indicative of large systems-level effects, rather than being limited to the vicinity of the target alone. It is noteworthy that significant variation was observed for several resistance proteins. 12 out of the 36 cytochromes present, five pumps (of 25), one HGT protein (of four) and three SOS proteins (of nine) were found to be upregulated in the expression profile corresponding to at least one of the seven drugs considered here. The study by Besra and co-workers [12] reports the expression levels of proteins in *M. tuberculosis *at the minimal inhibitory concentration (MIC) levels of the various drugs, which has been considered as the primary data for analysis. The study by Fu [13] reports the expression levels at much higher drug concentrations (5 *μ*g/mL for isoniazid, MIC = 0.2 *μ*g/mL). It must be noted that it is possible that the high concentrations may lead to abnormal expression levels, but has been considered here as an additional set since with higher dose, there may be a higher propensity for resistance to develop. Information about the variation in gene expression levels has been incorporated into the MAP-RES network as described in the Methods section. In essence, a scoring scheme has been used to weight the edges in the network such that the edges between two upregulated nodes get better (lower) weights than edges formed by one but not both upregulated nodes, which in turn are better than the weights for edges between nodes that are not upregulated at all. The number of datasets from independent experiments in which a given node is upregulated is also taken into account; higher the number, better the weights.

### Identifying resistance pathways

A shortest path refers to the minimum number of hops required to reach one node from another in the network, where traversal along each edge is considered as a hop. Since these are computed on the whole interactome, all paths for the source to sink sets of nodes have been automatically evaluated to find the shortest paths, for the derivation of MAP-RES network. Shortest paths indicate the most feasible path that can be taken by a protein to communicate with another. The concept of shortest paths has been well accepted in graph theory and is a commonly used metric in the analysis of various networks in fields ranging from transport and communication to speech recognition [14]. It seems reasonable to assume that a path of two hops would be preferred to a path of four hops to transmit the same information from the same source to the same destination. We have used a scoring scheme (as described in the Methods section) to score the edges occurring in these shortest paths. The first parameter used in the scoring scheme is that involving 'betweenness', a measure of centrality. Node centrality or 'node-hubness' is a parameter that has been used often to indicate the relative importance of a given node [15]. Here, we have extended the concept to the edges and have evaluated 'edge-hubness' or edge centrality, also referred to as 'betweenness' [16]. In other words, a high value of edge betweenness indicates that the given edge occurs several times in all the possible paths available to reach a destination from a given source and that the traversal along this edge is inevitable. Taking roads in a city as an analogy, this is equivalent to a particular road and not merely a particular junction being the main link between two other nodes and hence traversal along that road being inevitable. Fig. 2 illustrates some edge hubs (shown as thick lines) in the MAP-RES network. The second parameter in computing the scores is the expression information, as described in the Methods section. If both nodes of a given edge, which is also an edge-hub are upregulated, then that edge gets the best score. The values for edge frequency vary from 78 to 1. For example, the edge Rv3546 (FadA5)-(395)→ Rv3545c (Cyp125) occurs 78 times in the shortest paths that comprise the MAP-RES network. The final path score is the summation of the individual edge scores that have considered both edge frequency and upregulation information. Thus, in obtaining the final scores, the path length also features as a parameter. The individual edge scores range from values close to zero to 1.0. The path scores were found to range from 0.0048 to 4.0. Scores close to zero refer to a top ranked path (a low 'cost' path), with a very short path length, where all nodes in it are upregulated and all edges have high betweenness values, whereas a score such as 4.0 refers to the other extreme, a path of length four, that contains edges of low betweenness (appearing only once in the set of shortest paths) and none of its nodes being upregulated.

#### Paths to resistance from targets in MAP

**Figure 3:**
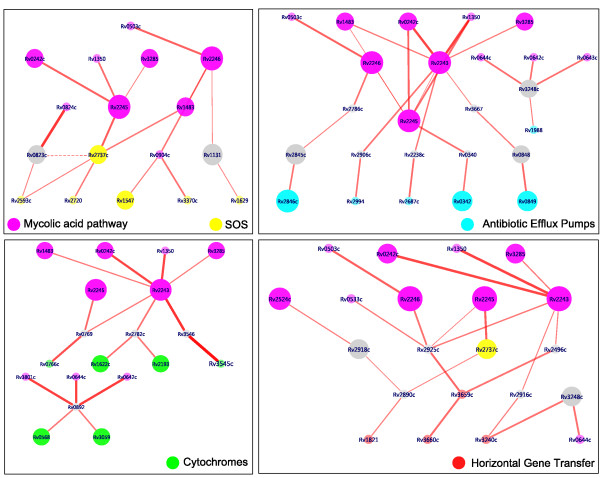
**Top scoring paths from MAP to each of the four resistance classes**. Nodes are labelled by their Rv IDs, as obtained from TubercuList. Nodes correspond to the individual proteins in the network while the edges indicate interactions between them. Each class of nodes is coloured differently as indicated. Grey nodes indicate those that do not belong to any of the marked classes. The nodes are sized in proportion to the number of MAP drugs that induce its upregulation. The thickness of an edge is proportional to the number of times a shortest path is traversed through that edge. The dotted edge is not a high-scoring path but is of significance, as discussed in the text.

The best ranked shortest paths to each of the four resistance mechanisms were identified as shown in Table 2 (for the complete list, see Additional File 3). High-scoring paths to each of the resistance mechanisms were observed from MAP (Fig. 3). However, paths to SOS proteins top the list, followed by paths to cytochromes, while paths to HGT and pumps were of much lower rank. Nodes and edges that occur most frequently in a given set of paths are considered as node and edge hubs. Top edge-hubs in MAP-RES are provided as supplementary material [see Additional File 4]. Several of the nodes in these edge hubs also happen to be top node hubs. Some such edge hubs are MmaA4 (Rv0642c) – Rv0892, FabD (Rv2243) – FadA5 (Rv3546), FadA5 (Rv3546) – Cyp125 (Rv3545c), Rv0049 – PcaA (Rv0470c), Rv0823c – DesA1 (Rv0824c), Acs (Rv3667) – Rv3779 and KasA (Rv2245) – RecA (Rv2737c).

**Table 2:** Top paths in the MAP-RES network

**Path**	**Score**
**SOS**	

**Rv0242c (FabG4) **-(276)→**Rv2245 (KasA) **-(596)→**Rv2737c (RecA)**	0.0102
**Rv0242c **-(276)→**Rv2245 **-(596)→**Rv2737c **-(44)→**Rv2720 (LexA)**	0.0727
Rv0904c (AccD3) -(241)→**Rv1547 (DnaE1)**	0.1000
Rv0824c (DesA1) -(162)→**Rv0823c **-(247)→**Rv2737c**	0.1344
**Rv0242c **-(276)→**Rv2245 **-(596)→**Rv2737c **-(96)→**Rv2593c (RuvA)**	0.1352
**Rv2246 (KasB) **-(400)→**Rv1131 (GltA1) **-(526)→Rv1629 (PolA)	0.1389

**CYTOCHROMES**	

**Rv2243 (FabD) **-(200)→Rv3546 (FadA5) -(395)→Rv3545c (Cyp125)	0.0174
**Rv0242c **-(436)→**Rv2243 **-(200)→Rv3546 -(395)→Rv3545c	0.0203
**Rv1350 (FabG2) **-(205)→**Rv2243 **-(200)→Rv3546 -(395)→Rv3545c	0.0230
**Rv1483 (FabG1) **-(121)→**Rv2243 **-(200)→Rv3546 -(395)→Rv3545c	0.0352
**Rv2243 **-(257)→Rv0769 -(558)→Rv0766c (Cyp123)	0.0643
**Rv0242c **-(436)→**Rv2243 **-(559)→Rv2782c (PepR) -(513)→**Rv1622c (CydB)**	0.0655
**Rv0242c **-(436)→**Rv2243 **-(257)→**Rv0769 **-(558)→**Rv0766c (Cyp123)**	0.0673
**Rv0242c **-(436)→**Rv2243 **-(559)→Rv2782c -(578)→**Rv2193 (CtaE)**	0.0738
Rv0643c (MmaA3) -(350)→Rv0892 -(350)→**Rv0568 (Cyp135B1)**	0.0956
Rv0643c -(350)→Rv0892 -(350)→**Rv3059 (Cyp136)**	0.0956

**ANTIBIOTIC EFFLUX PUMPS**	

**Rv2245 **-(565)→Rv0340 -(447)→**Rv0342 (IniA)**	0.0526
**Rv0242c **-(276)→**Rv2245 **-(565)→**Rv0340 **-(447)→**Rv0342**	0.0580
**Rv2243 **-(356)→Rv2238c (AhpE) -(569)→Rv2687c	0.0901
**Rv2243 **-(2)→**Rv2245 **-(565)→Rv0340 -(447)→**Rv0342**	0.1151
Rv0642c (MmaA4) -(350)→**Rv3248c (sahH) **-(486)→Rv3240c -(323)→Rv3239c	0.1365
Rv0642c -(350)→**Rv3248c **-(350)→Rv1988	0.1385
Rv1350 -(90)→**Rv2245 **-(596)→**Rv2737c **-(429)→Rv2882c (Frr) -(584)→Rv0783c (EmrB)	0.3169
**Rv2245 **-(369)→Rv1908c (KatG) -(350)→Rv1988	0.3833
**Rv0242c **-(276)→**Rv2245 **-(369)→Rv1908c -(350)→Rv1988	0.3888

**HORIZONTAL GENE TRANSFER**	

Rv0644c (MmaA2) -(350)→**Rv3248c **-(486)→Rv3240c (SecA1)	0.0812
**Rv2243 **-(202)→Rv2925c (Rnc) -(378)→Rv3659c	0.0913
**Rv2243 **-(202)→Rv2925c -(378)→Rv3659c -(63)→Rv3660c	0.1538
**Rv2245 **-(596)→**Rv2737c **-(514)→Rv2890c (RpsB) -(187)→Rv3240c	0.3715
**Rv2524c (Fas) **-(464)→**Rv2918c (GlnD) **-(380)→Rv2890c -(187)→Rv3240c	0.4833

The weighted score for each path is shown. The nodes upregulated by MAP drugs are indicated in bold typeface, while those that are upregulated by other drugs are underlined. The figures in parentheses indicate the edge weight; a lower edge weight indicates a higher confidence in the interaction. The edge weights range from 1 to 999. Lower edge weights are better. STRING scores range from 1 to 999. However, since edge weights are conventionally represented such that lower weights indicate better paths, the edge weights have been computed as 1000-STRING_score.

It is interesting to note that many of these edges can be attributed to metabolic linkages wherein the reactions involving the two proteins share a common metabolite (e.g. Rv0642 – Rv0892 or Rv0892 – Rv3801c). A few other edges could be attributed to adjacency of the genes in the genome whose transcription may be regulated by a common mechanism. A number of these proteins are also upregulated in the top paths (Figs. 2, 3). In some cases, the entire paths (i.e., all nodes in the path) were upregulated in one or more of the drugs, indicating the correlation of the identified paths with the observed expression profiles. A path from FabG4 (Rv0242c) → KasA (Rv2245) → RecA (Rv2737c), appears to be such a path, where all nodes are upregulated, making the flow of information from source to sink that of high propensity. Another interesting path is from DesA1 (Rv0824c) → Rv0823c → RecA (Rv2737c), which also has an alternate sub-path to RecA through RuvA (Rv2593c). In this path, besides RecA, Rv0823c is also upregulated (Fig. 3). Considering the individual functions of these molecules, it is easy to comprehend that the transcriptional regulator (Rv0823c) influenced by DesA1 (Rv0824c), triggers the activation of RecA (Rv2737c), which while itself a sink, also activates many proteins such as DnaE1 (Rv1547), important for DNA synthesis. All these proteins are upregulated in response to one or more of the MAP inhibitors. DesA1 was earlier identified as a potential anti-tubercular drug target [10], by virtue of its critical role in mycolic acid biosynthesis [17]. From the above observations, it is clear that targeting RecA or DnaE1 or Rv0823c together with DesA1 will be a good strategy to counter emergence of drug resistance. Thus, identification of these proteins opens up new possibilities, illustrating a new concept to prevent drug resistance.

#### Co-targets as a new concept

**Figure 4:**
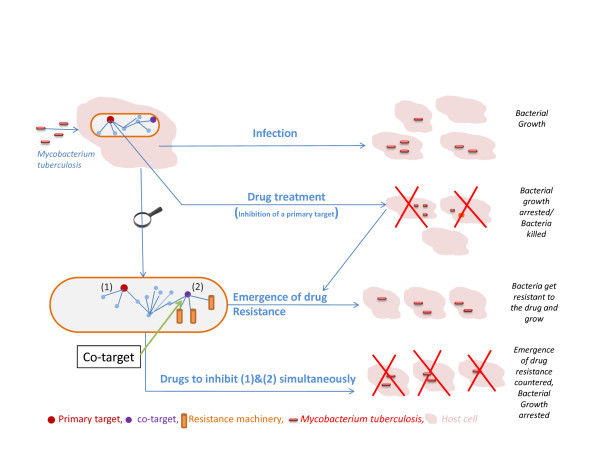
**Schematic diagram depicting the concept of 'co-targets'**. A schematic diagram depicting the concept of 'co-targets'. A bacterium upon infection under suitable conditions, leads to bacterial growth. Upon drug treatment however, the intended target of the drug, referred to as the 'primary target' is inhibited and bacteria are either killed or their growth arrested substantially. Over a course of time however, the remnant bacteria develop resistance to the administered drug, resulting in bacterial growth once again. Drug resistance develops by triggering the resistance machinery upon drug exposure. Communication to the resistance machinery from the drug target is established through channels (pathways identified in this study) in the protein-protein interaction network. Proteins important for mediating such communication are termed 'co-targets' and their simultaneous inhibition by suitable drug combinations along with the primary target, will help in preventing emergence of drug resistance, thus rendering the primary drugs useful again. (1) and (2) refer to the primary target and the co-target respectively, which should be considered as a pair (details in text).

To describe such proteins, we propose the term 'co-targets'. Co-targets refer to those proteins, which when inhibited simultaneously with a corresponding primary target, will help in reducing the emergence of resistance to the drug binding to that primary target. Co-targets should not be confused with ancillary or secondary targets, although there is no standard definition for the latter two terms. The concept is illustrated in Fig. 4. Ancillary targets have been referred to describe proteins through which a drug exerts additional beneficial effects, perhaps through auxiliary pharmacodynamic effects, such as that of benzodiazepines in bipolar disorders [18], whereas the term 'secondary target' (and a corresponding secondary drug) has been used to describe those proteins that aid in alleviating the side effects caused by a primary drug [19]. The term has also been used to refer to those targets that help in improving the bio-availability of the drug, albeit without any explicit consideration of drug resistance.

A co-target should always be considered as one of the two components forming a pair, where the other is a primary target. This is irrespective of the function of the protein when considered individually, since some co-targets could be primary targets themselves, but also turn out to be important in mediating resistance for another target. Thus, co-targets could have diverse functions, could be either essential or non-essential to the microbe, but necessarily have a strong influence in the network, to mediate information flow from a given target. The determining feature to be called a co-target is its ability to counter resistance for drugs modifying the corresponding primary target. Ancillary or secondary targets, on the other hand, usually refer to those proteins, which by themselves are not essential and do not have a critical physiological function for the survival of the cell in question, but help in modifying the properties of the drug intended for a given target. They have not been viewed in the context of drug resistance so far.

As many as 19 cytochrome proteins were found to be present in a tight cluster (Fig. 2), seven of them upregulated, connected to MAP through Rv0892, which is annotated as a probable monooxygenase. Some noteworthy pathways across the four classes are: (i) Rv2243 (FabD) → Rv3546 (FadA5) → Rv3545c (Cyp125) (cytochrome); (ii) Rv2524c (Fas) → Rv2918c (GlnD) → Rv2890c (RpsB) → Rv3240c, the latter a translocase implicated in HGT; (iii) Rv2245 (KasA) → Rv1908c (KatG) → Rv1988 (a methyltransferase annotated as an efflux pump); (iv) Rv2245 (KasA) → Rv2737c (RecA), Rv0904c (AccD3) → Rv1547 (DnaE1), both important for homologous recombination and DNA synthesis.

In fact, RecA (Rv2737c) and a few other proteins such as SecA1 (Rv3240c), SahH (Rv3248c), Rv0892 and MetK (Rv1392) occur in multiple resistance mechanisms, making their roles even more prominent in the emergence of resistance. Some of the MAP proteins such as KasA (Rv2245), FabD (Rv2243), which are upregulated, also appear to mediate multiple pathways across different resistance mechanisms. Proteins important for multiple resistance pathways could form ideal 'co-targets'. Of the top node and edge hubs in MAP-RES, it is of interest to note that nodes Rv0892 and Rv2243 are also among the top hubs in the entire STRING network, indicating their critical role in the *M. tuberculosis *interactome. The nodes and edges in the STRING, ranked based on their betweenness, are provided as supplementary material [see Additional File 5].

Table 2 also indicates shortest paths containing proteins upregulated in response to one or more of the four non-MAP drugs, used for obtaining the microarray data, along with the three MAP drugs discussed so far. It was observed that while paths to SOS still topped the list, many paths to cytochromes, pumps and HGT were found and contained a number of upregulated proteins, indicating that propensities for traversal of a path could vary from drug to drug.

#### Paths to resistance from other mycobacterial drug targets

Besides drugs such as isoniazid and ethionamide that target the MAP, several other drugs are also used for the treatment of TB. The best examples of these are rifampicin and ciprofloxacin. The known target of rifampicin is the B subunit of RNA polymerase (RpoB, Rv0667), while DNA gyrase (GyrA, Rv0006) is the target of ciprofloxacin and other members of the fluoroquinolone series. Other known targets of anti-tubercular drugs are cell wall biosynthesis proteins such as Alr (Rv3423c), DdlA (Rv2981c), Rv3792, EmbA (Rv3794) and EmbB (Rv3795) for cycloserine. Each of these targets and their parent pathways where possible, were individually considered as 'source' nodes and new sub-networks derived in each case, by computing the shortest paths to the previously defined set of 'sink' proteins.

Short high-scoring paths were observed from all targets examined, to the SOS response, involving many common nodes (e.g. DnaE1 (Rv1547), RecA (Rv2737)), whereas paths to other resistance mechanisms differed from target to target. Some edges in the SOS response (e.g. Rv2158c – Rv0631c) were common to paths from cell wall proteins and gyrase. From gyrase, SOS was the only predominant path, whereas from RpoB, paths to HGT and cytochromes were also among the top paths. For RpoB, many of the paths to SOS were mediated through Dcd (Rv0321), a DCTP deaminase. From cell wall proteins, paths to HGT and SOS top the list of high-scoring paths again, although there are also several paths to cytochromes. Paths to pumps were relatively fewer in number, in all cases. Of interest, however was a path to EfpA (Rv2846c), a transporter known to confer resistance to fluoroquinolones (Rv0006 → Rv0524 → Rv3065 → Rv2846c), rifampicin and isoniazid [6]. A higher scoring path to IniA (Rv0342) was observed through Rv0340 (conserved hypothetical protein), again agreeing well with previous reports based on transcription studies [20]. We also analysed paths from proteins KatG (Rv1908c), EthA (Rv3854c) and PncA (Rv2043c), all known to transform pro-drugs to the active drug species to the sink. It was interesting to observe that while EthA and PncA did not appear to have short paths to any of the resistance mechanisms, KatG had a direct interaction with an efflux pump (Rv1988, a probable methyltransferase, homologue of Erm37) and also several paths of two or fewer edges to all resistance mechanisms. In fact, studies on clinical isolates have shown mechanisms such as mutations in KatG, leading to loss of catalase activity, mutations in the promoter region of *inhA*, leading to its over-expression and mutations in InhA, leading to loss of affinity for isoniazid [3]. Paths to different resistance mechanisms for different drugs observed here, suggest that a given target may have a higher propensity for eliciting a specific mechanism of resistance, which when understood can be utilised to identify appropriate co-targets, to prevent the emergence of resistance.

Upon analysis of the network formed only by the upregulated proteins [see Additional File 6], for each of the seven drugs, we observe that the network density is much higher, over an order of magnitude in most cases, than that of the whole interactome. This indicates that the upregulated genes have a higher influence on each other and more importantly, their inter-relatedness is perhaps for a specific purpose. Such a purpose, if present, is non-obvious by their functional classes or by a common analysis (such as co-expression) of the microarray data, except in a few cases. However, when viewed in the context of the interaction networks, it becomes possible to analyse the outcome of such inter-relatedness. It is important to bear in mind that studies on a theoretically derived network such as this may have some limitations, due to missing out some important interactions not identified by any of the computational methods used or due to false positives that may be present in the network. However, with the care taken during the construction and the analysis, and the experimental support available in the literature for protein-protein interaction prediction [21,22], we believe that such errors if present may not be many and the used network still captures the interactions in the mycobacterial cell well. This study also serves as a framework to design experiments to verify the role of identified edge-hubs and co-targets. The new formulation of the problem of drug resistance in this study also serves as a starting point to understand the structural, biochemical and thermodynamic basis in detail, by which such communication is facilitated.

#### Support from experimental evidence as proof-of-concept

Reports in literature indicate that the main strategies for adaptation by the bacillus in response to these drugs are mutations [23], to reduce the binding of these drugs to the MAP target(s). This inherently implies that the MAP proteins have a streamlined mechanism to pass on the information of drug inhibition (or lack of mycolic acid production) to the SOS proteins involved in recombination and DNA biosynthesis. A time course microarray data over a period of a few weeks, from clinical samples, would ideally have been required to see the expression patterns where resistance has emerged. Nevertheless, the existing data provides a first glimpse of the possible routes that might lead to resistance.

The SOS regulon that includes RecA and LexA has been implicated in the response of other organisms to drugs that target cell wall metabolism [24]. The disruption of the SOS polymerases or prevention of LexA autoproteolysis have been suggested previously to be useful in limiting evolution of resistance [25]. Available experimental evidence also suggest that the inhibition of RecA might be a possible strategy to limit mycobacterial genomic evolution [26], also confirmed by the genomic stability of a natural *recA *mutant [27]. Wigle and Singleton have studied inhibitors for RecA, and propose that such inhibitors may be developed into novel adjuvants for antibiotic chemotherapy that moderate the development and transmission of antibiotic resistance genes [28].

## Conclusion

The analysis reported here has led to the identification of most probable routes that may be utilised to bring about resistance to a given drug. Identification of pathways to resistance is novel, providing another example of the remarkable usefulness of a systems-level analysis. Another interesting observation was that different targets seemed to have different propensities for drug resistance mechanisms. This strongly suggests that certain targets and therefore, drugs acting through those target proteins, will have a higher chance of the microbe developing resistance against them as compared to some others. This leads to the possibility of developing a new concept to assess the druggability of a target. To our knowledge, such information has not been used earlier in the identification or evaluation of drug targets.

We introduce the concept of 'co-targets', which forms a new rational strategy for combating drug resistance. A set of target-co-target pairs in *M. tuberculosis *have been identified. Given the rapid accumulation of various types of 'omics' data, including comprehensive views of protein-protein interactions, this type of analyses is becoming feasible for many pathogenic organisms. Our approach is also inherently generic, lending itself to be utilised in many drug discovery programmes.

## Methods

### Interactome network derivation

A proteome-scale interaction network of proteins in *M. tuberculosis *was derived from the STRING database, using only the 'high-confidence' and 'medium-confidence' data. The individual interactions as well as their confidence scores are provided as supplementary material [see Additional File 1].

### Curation of resistance genes

We curated a list of 74 genes in the *M. tuberculosis *genome based on resistance mechanisms from published literature [6,7,29-32] (Table 1). Available biological literature was scanned to obtain information about associations of individual proteins with drug resistance and assignments to Rv numbers in the *M. tuberculosis *H37Rv proteome were manually verified before including in the list.

### Betweenness

Betweenness is a centrality measure of a vertex within a graph [33]. For a graph G(V, E), with *n *vertices, the betweenness *C*_*B*_(*υ*) of a vertex *υ *is defined as

$$C_{B}(v)=\sum_{s\neq v\neq t\in V}\frac{\sigma_{st}(v)}{\sigma_{st}}$$

where *σ*_*st *_is the number of shortest paths from *s *to *t*, and *σ*_*st*_(*υ*) is the number of shortest paths from *s *to *t *that pass through a vertex *υ*. A similar definition for 'edge betweenness' was given by Girvan and Newman [16]. Betweenness was calculated for all nodes and edges in STRING, ranking them to obtain a list of hubs [see Additional File 5]. Essentially, betweenness is an indicator of how many shortest paths in the network a node or an edge is a part of. The computed betweenness is thus an absolute value, which depends upon number of shortest paths in the network and the frequency of occurrence of a given node/edge in the list of shortest paths from any node to any other node. Higher the betweenness, more important is the node/edge in the context of the network, since it forms a part of more shortest paths.

### Network analysis

Shortest paths from source to the curated set of resistance proteins (sink) were computed using Dijkstra's algorithm implemented in the MATLAB-Boost Graph Library (David Gleich; ). The algorithm is used to compute the distance (number of hops, in this case) and the predecessor (node in the path) for each of the vertices along the shortest path, from a particular vertex to every other vertex in the graph. Only those shortest paths that begin from a source node and terminate at a sink node were then obtained, to construct the MAP-RES network.

#### Scoring of paths

We added a weighting scheme to account for the frequency of an edge in the network, as well as to incorporate the upregulation information of the nodes forming a given edge. The weight of an edge ST is given as

$$w_{st}=\frac{1}{f_{st}(1+N_{s})(1+N_{t})}$$

where *f*_*st *_corresponds to the frequency of the edge between *s *and *t*, which is the number of times a given edge occurs in the set of paths, *N*_*s *_and *N*_*t *_refer to number of drugs (only drugs that target MAP are considered) for which node *s *and node *t *are upregulated respectively. Thus, the maximum possible score for an edge is unity, when the edge appears only once in the set of chosen paths and neither of the nodes forming the edge is upregulated. A path score was computed as the sum of the weighted scores for the edges in a path, from which the least scoring path corresponded to the highest rank. Path scores, consequently, vary from values close to zero (all edges have low scores), to values equal to path length (all edges have a score of unity). For the present network, path scores vary from 0.0048 to 4.0. The path score can also be thought of as the 'cost' of taking that particular path; lower the cost, more likely the path. Cytoscape [34] was used for network visualisations.

## Abbreviations

HGT: Horizontal Gene Transfer; MAP: Mycolic Acid Pathway; TB: Tuberculosis.

## Authors' contributions

NC generated the idea. KR and NC designed the experiments. KR carried out the computations. KR and NC analysed the data and wrote the manuscript.

## Supplementary Material

Additional File 1 **STRING network links.** These data, from the STRING database, form the basis for the network constructed in this study. Click here for file

Additional File 2 **MAP-RES network.** High-resolution image of the MAP-RES network, a portion of which has been indicated in Figure 2. Click here for file

Additional File 3 **Ranked MAP-RES paths.** This is a complete list of the paths that have been shown in Table 2. Click here for file

Additional File 4 **MAP-RES edge hubs.** A list of all the edges in MAP-RES, ordered by the number of times they appear in the network. Click here for file

Additional File 5 **STRING node and edge hubs.** Node and edge hubs in the interactome, constructed from STRING. Click here for file

Additional File 6 **Genes upregulated on drug treatment.** A comparison of the network parameters, for the sub-networks of upregulated proteins. Click here for file
