# Supplementary material for: Mycobacterium tuberculosis interactome analysis unravels potential pathways to drug resistance
Source: BMC Microbiol. 2008 Dec 23;8:234. doi: 10.1186/1471-2180-8-234 (PMC2649132; doi:10.1186/1471-2180-8-234)
Supplement: Additional File 2 — MAP-RES network. High-resolution image of the MAP-RES network, a portion of which has been indicated in Figure 2. [file 1471-2180-8-234-S2.pdf]

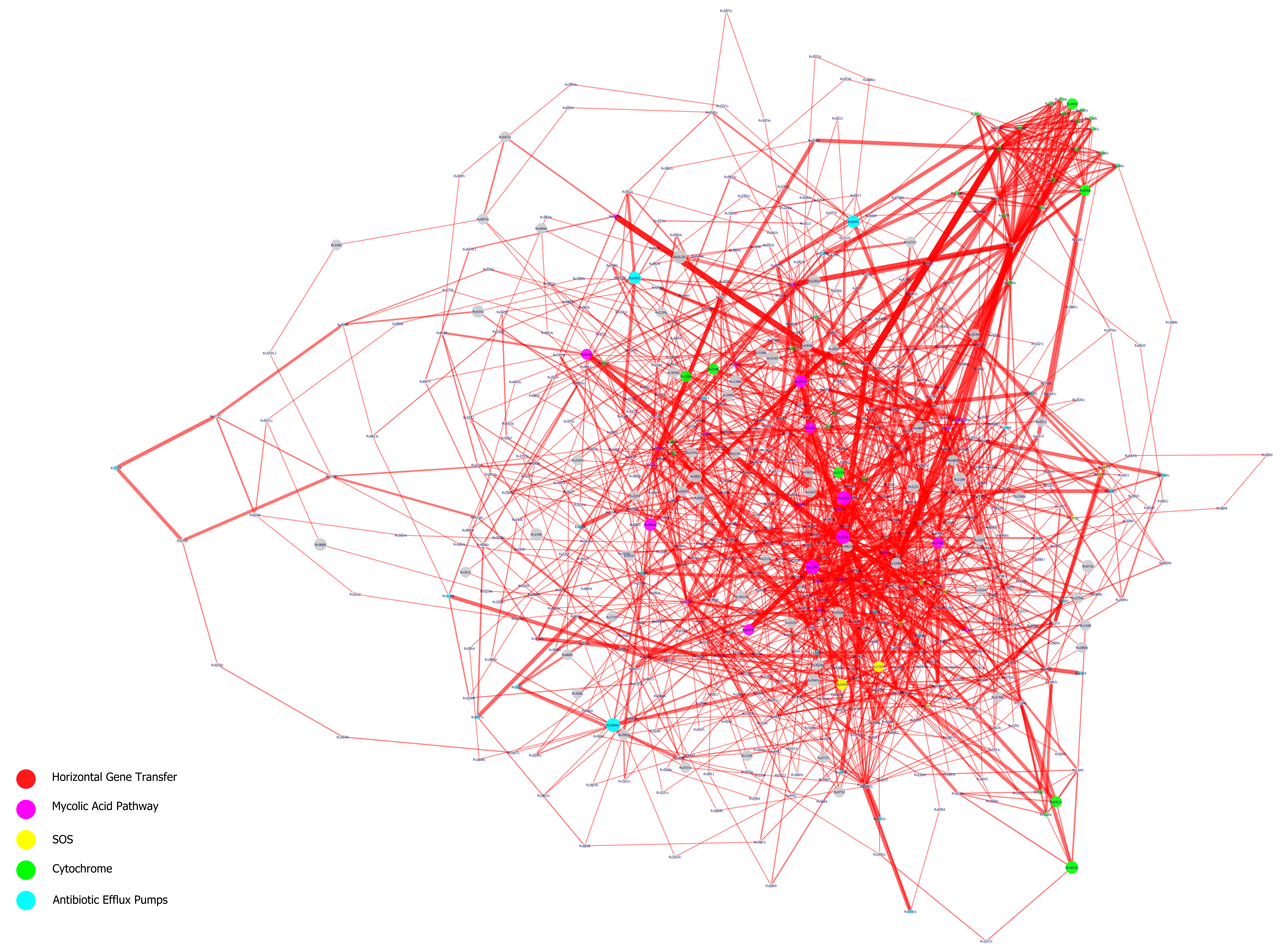
Horizontal Gene Transfer
Mycolic Acid Pathway
SOS
Cytochrome
Antibiotic Efflux Pumps
